# The Relationship Between Humor Styles and Forgiveness

**DOI:** 10.5964/ejop.v12i3.1012

**Published:** 2016-08-19

**Authors:** William Hampes

**Affiliations:** a Department of Psychology, Sociology, and Education, Black Hawk College, Moline, IL, USA; Department of Psychology, University of Western Ontario, London, Canada

**Keywords:** affiliative humor, self-enhancing humor, aggressive humor, self-defeating humor, and forgiveness

## Abstract

Research has shown that a factor in a victim’s forgiveness of an offender is the victim’s ability to make more positive, or at least less negative, attributions of the offender’s behavior and that perspective-taking can be a factor in facilitating that process. Self-enhancing humor has been found to be positively correlated with perspective-taking empathy and aggressive humor found to be negatively correlated with perspective-taking empathy. Therefore it was predicted that self-enhancing humor would be positively correlated with forgiveness and aggressive humor negatively correlated with forgiveness. The Humor Styles Questionnaire, the Absence of Negative and Presence of Positive subscales of the Forgiveness Scale, and the Forgiveness Likelihood Scale were administered to 112 college undergraduates. Self-enhancing humor was significantly and positively correlated with all of the forgiveness measures, aggressive humor and self-defeating humor were significantly and negatively correlated with some of the forgiveness measures and affiliative humor was not significantly correlated with any of the forgiveness measures. The results were interpreted in terms of previous findings for humor styles, perspective-taking empathy, depression, self-esteem and anxiety. Future research involving the extent to which other personality variables, such as perspective-taking empathy, mediate the relationship between self-enhancing humor and forgiveness was suggested.

Forgiveness, defined as the victim letting go of negative affect (e.g. hostility), negative cognitions (e.g. thoughts of revenge), and negative behavior (e.g. verbal aggression) toward the offender (Rye, Loiacono, Folck, Olszewski, Heim, & Madia, 2001) has been associated with the victim making more benign attributions of the offender’s behavior (Fincham, 2000; Hall & Fincham, 2006). Fincham, Paleari, and Regalia (2002) studied the relationship between marital quality, attributions, empathy and forgiveness and found that a victim’s positive relationship with an offender promotes more positive attributions of the offender spouse’s behavior, which in turn, promotes the victim’s forgiveness both directly and indirectly through affective reactions and emotional empathy. A similar model was postulated by Davis and Gold (2011), who found that remorse on the part of an offender reduced the victim’s attribution of behavioral stability (causing the victim to be less likely to believe that the offender would commit a transgression in the future) and thus increased empathy and forgiveness.

Besides marital quality and the offender’s remorse, perspective-taking could be another variable that promotes positive attributions of the offender’s behavior and thus forgiveness. Vescio, Sechrist, and Paolucci (2003) and Galinsky and Moskowitz (2000) found that those who engaged in more perspective-taking developed more positive attitudes towards members of an outgroup. Takaku (2001) found that those who engaged in perspective-taking were less likely to perceive the transgression of the offender as internal and stable and thus less likely to occur again. The results of another study (Arriaga & Rusbult, 1998) indicated that facilitating perspective-taking leads to more relationship-enhancing and fewer partner-blaming attributions. Takaku, Weiner, and Ohbuchi (2001) found that being better able to understand another’s perspective by recalling oneself as a wrongdoer increased the individual’s ability to forgive an offender. These studies are thus consistent with the positive correlation between perspective-taking ability and forgiveness (Rizkalla, Wertheim, & Hodgson, 2008).

There is considerable research on the relationship between humor and perspective-taking, in particular in regards to reducing stress by taking a more positive perspective of one’s situation. Martin (2007, p. 282) in his review of the research on humor, stress, and coping states: “Many authors have noted that humor, because it inherently involves incongruity and multiple interpretations, provides a way for individuals to shift perspective on a stressful situation, reappraising it from a new and less-threatening point of view”. One particular measure of humor, the Coping Humor Scale (Martin & Lefcourt, 1983, p. 1316), determines the extent to which a person copes with stress through the use of humor, including changing one’s perspective of a stressful situation to one that is less threatening (e.g. “I have often found that my problems have been greatly reduced when I tried to find something funny in them”, “I can usually find something to laugh or joke about in trying situations”). High scores on the Coping Humor Scale have been positively associated with taking a philosophical and detached view of problems (Fry, 1995), the conscious ability to successfully view problems from a more positive perspective (Kuiper, McKenzie, & Belanger, 1995), and viewing examinations as more as a challenge and less as a threat (Kuiper, Martin, & Olinger, 1993).

A study by Hampes (2010) has demonstrated that a particular type of humor, self-enhancing humor, is associated with the ability to take the perspective of another person, thus understanding better that person’s thoughts and feelings. According to Martin, Puhlik-Doris, Larsen, Gray, and Weir (2003) there are four types of humor: affiliative humor (the use of humor to amuse others, put others at ease and to improve relationships), self-enhancing humor (which enables a person to deal with stressful situations by taking a humorous perspective of them), aggressive humor (the inclination to use humor to put down or attack other people which involves teasing, ridicule, sarcasm, hostility, derision, or disparagement types of humor), and self-defeating humor (excessive self-disparaging humor to gain favor with others, laughing along with others when being disparaged or ridiculed, and going along with being the “butt” of others’ humor). According to Davis (1980) there are four different types of empathy: perspective-taking empathy, empathic concern, personal distress, and fantasy empathy. Perspective-taking empathy is the cognitive ability to understand another person’s viewpoint, thoughts and feelings. Empathic concern is how much a person feels compassion, warmth and concern towards individuals having negative experiences. Hampes (2010) found that self-enhancing humor was positively correlated with perspective-taking empathy, but was not correlated with empathic concern. On the other hand, he found that affiliative humor was positively correlated with empathic concern, but was not correlated with perspective-taking empathy. Aggressive humor was negatively correlated with both perspective-taking humor and empathic concern. Self-defeating humor was not correlated with either empathy scale.

The purpose of this study was to investigate the relationship between the four humor scales and forgiveness. It was predicted that self-enhancing humor would be positively correlated with forgiveness due to its association with perspective-taking empathy, which in turn is positively correlated with forgiveness (Hodgson & Wertheim, 2007; Konstam, Chernoff, & Deveney, 2001; Zechmeister & Romero, 2002). Since those who are high in self-enhancing humor tend to be high in perspective-taking empathy, a victim high in self-enhancing humor would presumably be better able to take a perspective of those who have offended them that allows for a more positive attribution of the offender’s behavior towards them than the perspective taken by those who aren’t high in this style of humor. This would seem to make it easier for those high in self-enhancing humor to forgive the offender than those who are lower in self-enhancing humor. It was predicted that aggressive humor, which is negatively correlated with both perspective-taking empathy and empathic concern, would be negatively correlated with forgiveness since those high in aggressive humor lack the perspective-taking empathy that would help them to make the less negative attributions of the offender that would lead to forgiveness.

## Method

### Participants

The participants were 112 students (39 men and 73 women) at a community college in the Midwestern United States. They ranged in age from 17 to 57 (*M* = 22.82, *SD* = 7.96).

### Procedure

Students in six psychology classes were asked to participate and those who volunteered were included in the sample. Participants completed the Humor Styles Questionnaire, the Forgiveness Scale and the Forgiveness Likelihood Scale in their respective classes after completing a consent form which informed them of what their participation would involve and assuring them of confidentiality and the voluntary nature of their participation. The study was previously approved by the college’s Institutional Review Board.

### Materials

#### Forgiveness Scale

The Forgiveness Scale includes fifteen items divided into an Absence of Negative subscale with ten items stating negative feelings, thoughts and behaviors towards the offender (e.g. “I can’t stop thinking about how I was wronged by this person”) and a Presence of Positive subscale with five items reflecting positive feelings, thoughts and behaviors towards the offender (e.g. “I have been able to let go of my anger toward the person who wronged me”) that asks participants how they have responded to a person who has wronged or mistreated them. Each item has five response options in a Likert-type format (1 = Strongly Disagree to 5 = Strongly Agree). Validity was established with significant correlations for both subscales in the expected direction with measures of religiousness, hope, trait and state anger, spiritual and religious well-being and the Enright Forgiveness Inventory. The test-retest reliability was .76 for both the Absence of Negative and the Presence of Positive subscales and .80 for the total scale (Rye et al., 2001). The Cronbach alpha for the current sample was .84 for the total scale, .80 for the Absence of Negative subscale and .73 for the Presence of Positive subscale.

#### Forgiveness Likelihood Scale

The Forgiveness Likelihood Scale has ten items (e.g. “Your significant other has a ‘one night stand’ and becomes sexually involved with someone else. What is the likelihood that you would choose to forgive your significant other?”, “A stranger breaks into your house and steals a substantial sum of money from you. What is the likelihood that you would choose to forgive the stranger?”) that asks the participants how likely they would be to forgive someone in an imagined scenario. Each item has five response options in Likert-type format (1 = Not at all Likely to 5 = Extremely Likely). Validity was supported with significant correlations in the expected direction with religiousness, religious well-being, trait anger and the Enright Forgiveness Inventory. Each of the two subscales of the Forgiveness Scale was significantly correlated with the Forgiveness Likelihood Scale. The test-retest reliability of the Forgiveness Likelihood Scale was .81. (Rye et al., 2001). In the current study the Forgiveness Likelihood Scale was significantly correlated with the total Forgiveness Scale (*r* = .41, *p* < .001) and the Presence of Positive subscale (*r* = .43, *p* < .001) and Absence of Negative subscale (*r* = .28, *p* < .01) of the Forgiveness Scale. The Cronbach alpha for the Forgiveness Likelihood Scale for the current sample was .85.

#### Humor Styles Questionnaire

The Humor Styles Questionnaire has 32 items. Each scale, affiliative humor (e.g. “I laugh and joke a lot with my closest friends”), self-enhancing humor (e.g. “If I am feeling depressed, I can usually cheer myself up with humor”), aggressive humor (e.g. “If I don’t like someone, I often use humor or teasing to put them down”), and self-defeating humor (e.g. “I will often get carried away in putting myself down if it makes my family or friends laugh”) has eight items. Each item has seven response options in Likert-type format (1 = Totally Disagree to 7 = Totally Agree). The convergent validity for the affiliative humor scale was established by significant positive correlations with Extraversion on the NEO PI-R and Miller Social Intimacy Scale. Discriminant validity for the self-enhancing scale was indicated by a significant negative correlation with scores for Neuroticism on the NEO PI-R, and convergent validity was indicated with significant positive correlations with the Humor Coping subscale of the Coping Orientation to Problems Experienced Scale and the Coping Humor Scale. Convergent validity for the aggressive humor scale was estimated by a significant positive correlation with scores on the Cook-Medley Hostility Scale. Discriminant validity for the self-defeating humor scale was supported by significant negative correlations with the Rosenberg Self-Esteem Scale and with the Index of Self-Esteem (Martin et al., 2003). The Cronbach alphas for the humor styles for the current study were affiliative humor (.65), self-enhancing humor (.84), aggressive humor (.72), and self-defeating humor (.79).

## Results

Product-Moment correlations between each of the four humor styles with the Forgiveness Likelihood Scale, the total Forgiveness Scale, and the Absence of Negative and the Presence of Positive subscales of the Forgiveness Scale were computed (see Tables 1 and 2). In addition, four multiple regression analyses were conducted, with the four humor styles as the predictor (independent) variables and the Forgiveness Likelihood Scale, the total Forgiveness Scale, and the Absence of Negative and the Presence of Positive subscales of the Forgiveness Scale as the dependent variables (see Tables 1 and 3).

**Table 1:** Means and Standard Deviations for the Four Humor Styles, the Forgiveness Likelihood Scale, the Total Forgiveness Scale, and Absence of Negative and Presence of Positive Subscales of the Forgiveness Scale

Scale	*M*	*SD*
Affiliative Humor	47.04	5.85
Self-Enhancing Humor	39.61	9.13
Aggressive Humor	28.59	7.85
Self-Defeating Humor	27.00	8.80
Forgiveness Likelihood Scale	25.19	7.57
Total Forgiveness Scale	51.81	8.88
Presence of Positive Subscale	14.96	4.11
Absence of Negative Subscale	36.82	6.40

**Table 2:** Pearson-Product Moment Correlations Between the Four Humor Styles and the Four Forgiveness Measures

Scale	Humor Styles			
	Aff	SE	Agg	SD
FLS	.06	.29**	-.18	.09
TFS	.09	.34***	-.25**	-.31***
FSPP	.02	.25**	-.27**	-.22*
FSAN	.12	.33***	-.18	-.27**

*Note.* Aff = Affiliative Humor; SE = Self-Enhancing Humor; Agg = Aggressive Humor; SD = Self-Defeating Humor; FLS = Forgiveness Likelihood Scale; TFS = the total Forgiveness Scale; FSPP = the Forgiveness Scale, Presence of Positive subscale; FSAN = the Forgiveness Scale, Absence of Negative subscale.

*p < .05. **p < .01. ***p < .001.

The positive correlations between self-enhancing humor and the total Forgiveness Scale (*r* = .34, *p* < .001), both the Absence of Negative (*r* = .33, *p* < .001) and Presence of Positive (*r* = .25, *p* < .01) subscales of the Forgiveness Scale and The Forgiveness Likelihood Scale (*r* = .29, *p* < .01) were all significant. None of the correlations between affiliative humor and the forgiveness measures, albeit positive, were significant. The correlations between aggressive humor and the total Forgiveness Scale (*r* = -.25, *p* < . 01) and the Presence of Positive subscale of the Forgiveness Scale (*r* = -.27, *p* < .01) were significant, but those between aggressive humor and the Forgiveness Likelihood Scale and the Absence of Negative subscale of the Forgiveness Scale were not. The negative correlations between self-defeating humor and the total Forgiveness Scale (*r* = -.31, *p* < .001), the Absence of Negative subscale of the Forgiveness Scale (*r* = -.27, *p* < .01) and Presence of Positive subscale of the Forgiveness Scale (*r* = -.22, *p* < .05), but not that between self-defeating humor and the Forgiveness Likelihood Scale, were significant.

**Table 3:** Multiple Regression Analyses With Scores Involving the Forgiveness Likelihood Scale and the Forgiveness Scale (Dependent Variables) and the Four Humor Styles (Predictor, Independent Variables)

Scale	Regression Betas for Humor Styles				*R*	*F* (4,111)
	Aff	SE	Agg	SD		
FLS	-.05	.30**	-.24*	.17	.38	4.41**
TFS	-.06	.36***	-.17	-.26**	.49	8.33***
FSPP	-.09	.28**	-.21*	-.16	.40	5.09***
FSAN	-.02	.33***	-.09	-.24**	.43	6.22***

*Note.* Aff = Affiliative Humor, SE = Self-Enhancing Humor, Agg = Aggressive Humor, SD = Self-Defeating Humor, FLS = Forgiveness Likelihood Scale, TFS = the total Forgiveness Scale, FSPP = the Forgiveness Scale, Presence of Positive subscale, FSAN = the Forgiveness Scale, Absence of Negative subscale.

*p < .05. **p < .01. ***p < .001. (Note that the Beta for aggressive humor predicting the Forgiveness Likelihood Scale taken to three decimal places was -.235, just barely missing the .01 level, whereas the Beta for self-defeating humor predicting the Absence of Negative subscale taken to three decimal places was -.243, just barely reaching the .01 level).

Self-enhancing humor positively and significantly predicted the total Forgiveness Scale (beta = .36, *p* < .001), both the Absence of Negative (beta = .33, *p* < .001) and Presence of Positive (beta = .28, *p* < .01) subscales of the Forgiveness Scale and the Forgiveness Likelihood Scale (beta = .30, *p* < .01). Affiliative humor did not significantly predict any of the forgiveness scales or subscales. Aggressive humor significantly negatively predicted the Forgiveness Likelihood Scale (beta = -.24, *p* < .05) and the Presence of Positive subscale of the Forgiveness Scale (beta = -.21, *p* < .05), but did not significantly predict the total Forgiveness Scale or the Absence of Negative subscale of the Forgiveness Scale. Self-defeating humor significantly negatively predicted the total Forgiveness Scale (beta = - .26, *p* < .01) and the Absence of Negative subscale (beta = -.24, *p* < .01) of the Forgiveness Scale, but did not significantly predict the Forgiveness Likelihood Scale or the Presence of Positive subscale of the Forgiveness Scale.

## Discussion

That self-enhancing humor positively and significantly predicted and was significantly and positively correlated with the four measures of forgiveness is entirely consistent with the positive correlation of self-enhancing humor with perspective-taking empathy. That aggressive humor negatively and significantly predicted two of the four measures of forgiveness and was significantly negatively correlated with two of them is partially consistent with the negative correlations of aggressive humor with perspective-taking empathy. However, affiliative humor was not correlated with any measure of forgiveness. The key to this could be that although affiliative humor is positively correlated with empathic concern, it is not correlated with perspective-taking empathy (Hampes, 2010). In the Zechmeister and Romero (2002), Hodgson and Wertheim (2007) and Konstam, Chernoff, and Deveney (2001) studies the correlations between perspective-taking empathy and forgiveness were higher than those between empathic concern and forgiveness and the correlations between empathic concern and forgiveness in the Zechmeister and Romero (2002) and Hodgson and Wertheim (2007) studies and between empathic concern and one measure of forgiveness in the Konstam, Chernoff, and Deveney (2001) study were not significant at the .05 level. It would seem that the cognitive trait of perspective-taking empathy is a more crucial determinant of forgiveness than the emotional trait of empathic concern. This is consistent with the models of Fincham, Paleari, and Regalia (2002) and Davis and Gold (2011), which postulate that positive attributions lead to *situational* emotional empathy for the offender which leads to forgiveness. To be able to develop those positive attributions it tends to be helpful for the victim to be able to be effective in perspective-taking (Arriaga & Rusbult, 1998; Galinsky & Moskowitz, 2000; Takaku, 2001; Takaku, Weiner, & Ohbuchi, 2001; Vescio, Sechrist, & Paolucci, 2003). The trait of empathic concern, on the other hand, involves a person having compassion for other individuals because of those other individuals’ psychological or physical pain. In the case of a victim who has been hurt by an offender, the victim’s personality trait of empathic concern is not directly relevant since it is the victim who has been hurt, not the other individual, in this case the other individual being the offender. This would seem to lessen the effectiveness of the victim’s trait of empathic concern for leading to forgiveness. In fact, individuals’ empathic concern may make them less likely to forgive the offender when they empathize with other victims of the offender. Fredrickson (2003) has found that negative emotions narrow an individual’s mental focus. By feeling the emotional pain and anger of a victim an individual’s perspective of the offender could be constricted, making it harder for that individual to make positive attributions of the offender’s behavior.

Although self-defeating humor negatively predicted three forgiveness measures (two significantly) and was significantly negatively correlated with three of them, it did not significantly predict nor was significantly correlated with the Forgiveness Likelihood Scale. In fact, self-defeating humor was positively correlated with and positively predicted the Forgiveness Likelihood Scale, albeit not significantly. This could be due to the fact that the items on the Forgiveness Likelihood Scale represent hypothetical situations while the Forgiveness Scale involves items derived from actual situations in the participant’s life. Those high in self-defeating humor tend to be psychologically vulnerable, given the positive correlation between self-defeating humor with depression and anxiety and negative correlation with self-esteem (Kuiper, Grimshaw, Leite, & Kirsh, 2004; Martin et al., 2003) and positive correlation with anxious attachments (Cann, Norman, Welbourne, & Calhoun, 2008; Kazarian & Martin, 2004; Saroglou & Scariot, 2002). Because of this vulnerability they might find the situations that happened to them in the items of the Forgiveness Scale, particularly the ten items that make up the Absence of Negative subscale (e.g. “I become depressed when I think of how I was mistreated by this person”, “I avoid people and/or places because they remind me of the person who wronged me”) too painful for forgiveness. However, it could be easier to forgive someone in the situations in the Forgiveness Likelihood Scale since they may never have experienced them and are hypothetical.

The current study has several limitations. First of all it is correlational in nature. It is not clear whether the humor style affects forgiveness, forgiveness affects the humor style, or some third variable is affecting both. Secondly, the participant sample study was somewhat homogenous in terms of age. Perhaps the relationship between the humor styles and forgiveness could change as a person matures. Thirdly the sample size could be larger. Finally, the Cronbach alpha for affiliative humor (.65) is relatively low. Replicating this study with a sample size at least twice as large and with a higher alpha for affiliative humor could strengthen the conclusions drawn from it.

There are several directions that future research on humor styles and forgiveness could take based on the limitations of the current study. Studies could be done teasing out the causal relationships between humor styles and forgiveness. Researchers could possibly train participants to use self-enhancing humor better and then see if their forgiveness scores are higher than those of a control group. Another study could use the method developed by van Oyen Witvliet, Ludwig, and Vander Laan (2001) to ask participants to imagine forgiveness toward real-life offenders and then determine if this affected their humor styles.

Longitudinal studies could also be conducted to see if the relationship between humor styles and forgiveness changes as one gets older, studying a group of participants as they age from adolescence to adulthood. If this would be too time-consuming or impractical, a cross-sectional study with participants of different ages could still illuminate how the relationship between humor styles and forgiveness changes as one grows older.

Future research also is needed to determine how much of the variance for forgiveness is accounted by variables that are correlated with self-enhancing humor. Dyck and Holtzman (2013), Páez, Mendiburo Seguel, and Martínez-Sánchez (2013) and Ruch and Heintz (2013) have found that the incremental validity of the four humor styles are low once other personality variables are accounted for. Neuroticism is negatively correlated with and agreeableness is positively correlated with self-enhancing humor (Martin et al., 2003) and forgiveness (McCullough & Hoyt, 2002). That could possibly mean that the amount the variance of forgiveness predicted by self-enhancing humor could be considerably reduced once neuroticism and agreeableness, and other variables such as perspective-taking empathy, are accounted for.
